# The inhibition of tamoxifen on UGT2B gene expression and enzyme activity in rat liver contribute to the estrogen homeostasis dysregulation

**DOI:** 10.1186/s40360-022-00574-6

**Published:** 2022-05-31

**Authors:** Zhixiang Hao, Jiahao Xu, Han Zhao, Wei Zhou, Zhao Liu, Shiqing He, Xiaoxing Yin, Bei Zhang, Zhongjian Wang, Xueyan Zhou

**Affiliations:** 1 Jiangsu Key Laboratory of New Drug Research and Clinical Pharmacy, College of Pharmacy, Xuzhou Medical University, 209 Tongshan Road, Xuzhou, 221004 China; 2 Department of Pharmacy, Xuzhou First People’s hospital, 221000 Xuzhou, China; 3 Department of Thyroid and Breast Surgery, the Affiliated Hospital of Xuzhou Medical University, Xuzhou, China; 4 Department of Obstetrics and Gynecology, Xuzhou Central Hospital, Xuzhou Clinical School of Xuzhou Medical University, Xuzhou, China

**Keywords:** Breast cancer, Tamoxifen, Estrogens, Estrogen metabolizing enzyme, UGT2B

## Abstract

**Background:**

Tamoxifen treatment may induce dysregulation of estrogen homeostasis, leading to the occurrence of related adverse reactions. However, the potential mechanisms are still unclear. The purpose of the present study was to uncover whether tamoxifen treatment would act on estrogen metabolism-related biological enzymes and the regulatory effect on estrogen homeostasis to clarify the key factors and potential mechanisms of adverse reactions caused by long-term use of tamoxifen.

**Method:**

Female SD rats were administrated with tamoxifen CMC-Na solution (p.o.) once daily for four weeks and then housed at room temperature. Serum, breast, liver, uterus, and ovarian tissues were obtained, and the effects of tamoxifen administration on estrogen homeostasis, the expression, and activity of estrogen metabolic enzyme were evaluated.

**Results:**

Compared with the control group, the estrogen homeostasis was disturbed and the expression and activity of UGT2B1 (homology with human UGT2B7) were significantly reduced in the rats administrated with tamoxifen. The inhibitory effect of tamoxifen on UGT2B7 was dominated by hydrophobic and π-π stacking interactions, resulting in a concentration-dependent inhibition of UGT2B7 activity by tamoxifen and the imbalance of ligand-activated transcription factors, leading to abnormal regulation of UGT2B and disturbance of estrogen homeostasis, which in turn led to adverse reactions of tamoxifen.

**Conclusion:**

We established links between estrogen metabolism and tamoxifen administration and we proposed that the UGT2B inhibition was involved in the disturbance of estrogen homeostasis and the occurrence of tamoxifen-related adverse reactions.

**Supplementary Information:**

The online version contains supplementary material available at 10.1186/s40360-022-00574-6.

## Background

Breast cancer is one of the three most commonly diagnosed malignancies and the second leading cause of cancer-related death for women. 70% of breast cancers are estrogen receptor (ER) - positive breast cancers [1]. Tamoxifen, one of the first generation of selective estrogen receptor modulators (SERMs), behaves as an estrogen receptor antagonist in breast tissue and is used to prevent and treat estrogen ER-positive breast cancer [2–4]. Clinical trial results indicate that the extension of tamoxifen adjuvant therapy from 5 to 10 years reduces breast cancer recurrence [5, 6].

Despite these recognized benefits, the impact of tamoxifen on the imbalance of estrogen homeostasis and estrogen-related metabolic diseases is gaining more attention. Swerdlow et al. discovered that extending tamoxifen treatment for more than five years would increase the risk of endometrial cancer [7]. Mortimer et al. found that most women (78%) reported hot flashes, and 69% of those reporting hot flashes also reported night sweats among 864 women taking tamoxifen [8]. Cathcart et al. demonstrated that a 15% incidence of clinical depression related to tamoxifen, compared to 3% in women not placed on tamoxifen in the study of 301 women with breast cancer [9]. These studies suggest that tamoxifen treatment may induce dysregulation of estrogen homeostasis and related adverse reactions. However, the regulatory role of tamoxifen on estrogen homeostasis and the potential mechanisms are still unclear.

**Fig. 1:**
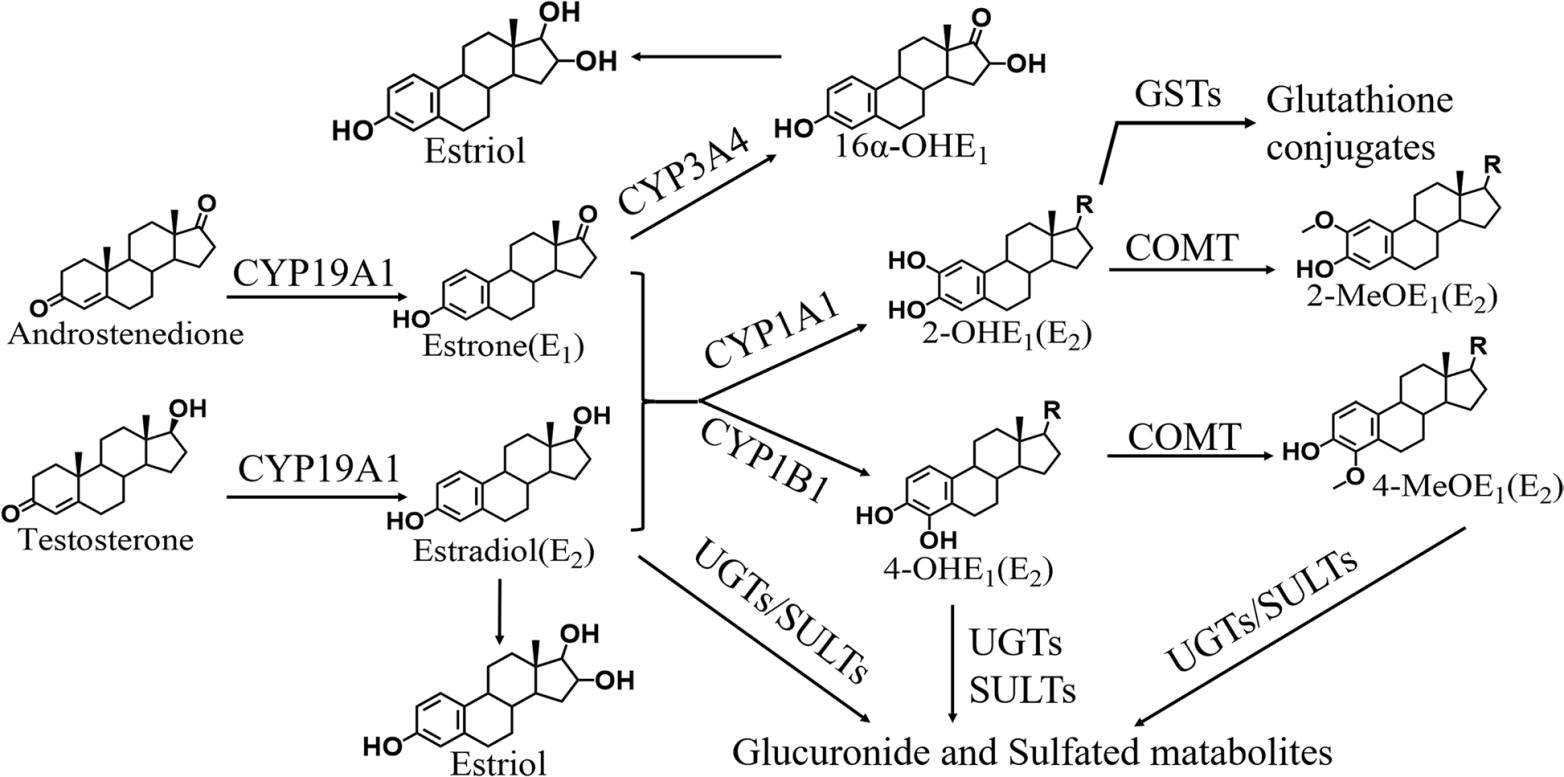
Estrogen active substance composition. The cited estrogen metabolites can undergo an additional degradation step by conjugation, either by glucuronidation, sulfation, or methylation, etc.

Estrogen homeostasis plays an important role in many physiological processes, including regulation of energy metabolism, and sexual development. A growing body of research suggests that once estrogen balance is disrupted, abnormal accumulation of estrogen is an important factor in the development of breast cancer [10]. And the metabolism of estrogen is closely linked to metabolic enzymes. As shown in Fig. 1, estrogen homeostasis is regulated by estrogen-related metabolic enzymes. Aromatase (CYP19), encoded by the CYP19A1 gene, is the crucial rate-limiting enzyme catalyzing the conversion of androgens to estrogens [11]. Estrone (E1) and estradiol (E2) are metabolized by the competitive pathways involving irreversible hydroxylations catalyzed by the NADPH-dependent cytochrome P450 (CYP) enzymes, including CYP1A1 and CYP1B1 [12]. E1 and E2 are hydroxylated at position C2, C4, and C16 and are converted to catechol estrogens 2-hydroxyestrogens (2-OHE1/E2), 4-hydroxyestrogens (4-OHE1/E2), and 16α-hydroxyestrone (16α-OHE1) [13, 14]. Estriol (E3) is produced by the hydroxylation of E2 or 16α-OHE1. Catechol estrogens are further metabolized (methylated) to methoxyestrogens (2-MeOE1/E2, 4-MeOE1/E2) by catechol-O-methyltransferase (COMT) enzyme [15].

In addition to methylation, parent estrogens and catechol estrogens are also conjugated with glucuronic acid by hepatic phase II enzymes UDP-glucuronosyltransferase enzymes (UGTs). UGTs are a superfamily located primarily in the endoplasmic reticulum of cells that detoxify a diverse range of xenobiotics and endogenous compounds [16]. In our previous study, we have verified that UGT contributes to estrogen elimination, and their glucuronidation capacity influences the estrogen signaling pathway and the pathogenesis of breast cancer [17]. UGT2B7 may play a key role in the elimination of estrogen. According to previous studies, the human UGT2B7 isoform does not exist in rat, and therefore the homologue UGT2B1 was studied in rat as a surrogate for the human UGT2B7 [18, 19]. It had been reported that tamoxifen had an inhibitory effect on the activity of CYP2C enzymes, but it can induce CYP3A4 on the transcript level [20]. The inhibition of aromatase CYP19A1 by Endox [21] (tamoxifen metabolite) and the gene induction of CYP3A4 by tamoxifen were also reported [22]. However, it is still unknown that whether tamoxifen also affects other estrogen metabolizing enzymes involved in estrogen homeostasis regulation.

Above the analysis, estrogen, which is the primary sex hormone responsible for the developing and modulation of the female reproductive system, is closely associated with adverse reactions to tamoxifen. To date, the role and mechanism of tamoxifen in regulating estrogen homeostasis are still unclear. Therefore, the purpose of the present study is to uncover whether tamoxifen treatment would act on estrogen metabolism related biological enzymes and the regulatory effect on estrogen homeostasis.

## Methods

### Materials

Estrone(E1), 17β-estradiol(E2), estriol(E3), 2-hydroxyestrone(2-OHE1), 2-hydroxyestradiol(2-OHE2), 2-methoxyestrone(2-MeOE1), 2-methoxyestradiol(2-MeOE2), 4-hydroxyestrone(4-OHE1), 4-hydroxyestradiol(4-OHE2), 4-methoxyestrone(4-MeOE1), 4-methoxyestradiol(4-MeOE2), 16α-hydroxyestrone(16α-OHE1), d5-E2, dansyl chloride, Vitamin C, naloxone, naloxone-glucuronide, D-Saccharic acid 1,4-lactone monohydrate and UGPGA were supplied by Sigma-Aldrich (St. Louis, USA) and Steraloids (Rhode, USA).

Tamoxifen was obtained from Aladdin (Shanghai, China). Alamethicin was obtained from J&K Scientific. TRIzol® and PrimeScript RT Reagent Kit were purchased from TaKaRa Bio-technology Co., Ltd. (Beijing, China). Human liver microsomes (HLMs) were acquired from Rild Liver Disease Research Co., Ltd (Shanghai). All analytical solvents were of liquid chromatography (LC) - grade and were obtained from Sigma-Aldrich (St. Louis, USA) or Merck (Darmstadt, Germany).

### Working solutions and buffers

The standard samples of 12 estrogens (E1, E2, E3, 2-OHE1, 2-OHE2, 2-MeOE1, 2-MeOE2, 4-OHE1, 4-OHE2, 4-MeOE1, 4-MeOE2, 16α-OHE1) and their metabolites and a stable isotope-labeled internal standard compound d5-E2 were accurately weighed and dissolved in methanol containing 0.1% Vitamin C. The single standard stock solution of 1 mM was prepared and stored at − 20 °C and kept away from light.

Before use, an equal volume of each standard stock solution was mixed evenly and diluted with methanol containing 0.1% Vitamin C to make a series of standard working solutions of specific concentration; The d5-E2 stock solution was diluted with methanol containing 0.1% Vitamin C to prepare 50 nM internal standard solution.

Tamoxifen solution (4 mg/ml) was prepared by dissolving the pure compound in 0.5% CMC-Na solution and stored at − 4 °C.

### Animals and treatment

Four-week-old female Sprague-Dawley rats were purchased from SIPPR-BK Lab Animal Co., Ltd. (Shanghai, China) and raised in a humidity-controlled room with free access to food and water. The temperature of the facility was maintained at 23 ± 2 °C with a 12:12-light-dark cycle. All animal studies were approved by the Animal Ethics Committee of Xuzhou Medical University and have been carried out in accordance with the Declaration of Helsinki. After one week of acclimatization, rats were randomly divided into the following two groups: Control group (*n* = 10) and Tamoxifen group (*n* = 10). Briefly, Control group included rats administered with CMC-Na p.o. once daily for four weeks; Tamoxifen group included rats administered with tamoxifen at a concentration of 20 mg/kg p.o. once daily that was dissolved in CMC-Na solution for 4 weeks. At the end of the experimental period (4 weeks), serum, mammary tissues, liver tissue, uterine tissue, and ovary tissues from animals were collected and frozen at − 80 °C for future analyses.

### Quantitative reverse transcriptase PCR (real-time PCR)

Total tissue extraction was performed using TRIzol® and cDNA was synthesized using the PrimeScript RT Reagent Kit according to the manufacturer s protocol. Quantitative reverse transcriptase PCR analysis was performed using the LightCycler®480II(Roche, Switzerland) to determine mRNA expression by the method described previously. Sequences of primer are listed in Table S1.

### Western blot analyses

Standard procedures performed western blotting. CYP1A1 (BS6575, Bioworld, USA), CYP1B1 (DF6399, Affinity Biosciences, USA), UGT1A (4371S, Cell Signaling Technology, Inc., USA), UGT2B (Ab113433, Abcam, UK), UGT2B7 (DF12140, Affinity Biosciences, USA) and GAPDH (AP0066, Bioworld, USA) primary antibodies were used individually for immunodetection. The intensities of the bands were quantified using Odyssey Sa (LICOR, USA).

### Preparation of S9

Take the mammary gland and liver tissues of experimental animals and place them in a pre-cooled PBS buffer to make 20% tissue homogenate. After centrifugation at 1000 g, the cell debris in the lower layer was removed, and the supernatant was aliquoted into 1.5 ml Eppendorf tubes. After centrifugation at 9000 g for 20 minutes, the supernatant was S9. The protein content of the obtained S9 was measured with the BCA kit and then placed in a − 80 °C for later use.

### UGT2B7 activity detection

The probe substrate incubation method was used to detect the activity of UGT2B7 [23]. The 200 μL incubation reaction system should include 50 mM Tris-HCl buffer (pH 7.4), 10 mM MgCl2, 6 mM D-Saccharic acid 1,4-lactone monohydrate, 25 μg/ml alamethicin, 3 mM UDPGA, HLMs (0.1 mg/ml), and 1 μL naloxone. After incubation at 37 °C for 30 min, 600 ul acetonitrile was added into naloxone incubation reaction system to terminate the reaction by precipitating the protein. The HPLC determined the corresponding metabolites.

### Concentration-dependent inhibition and time-dependent inhibition of tamoxifen on UGT2B7 activity

The 200 μL incubation reaction system should include 50 mM Tris-HCl buffer (pH 7.4), 10 mM MgCl2, 6 mM D-Saccharic acid 1,4-lactone monohydrate, 25 μg/ml alamethicin, 3 mM UDPGA, HLMs (0.1 mg/ml), 1 μL tamoxifen and 1 μL naloxone, a selective probe substrate. For the concentration-dependent inhibition experiment of tamoxifen on UGT2B7 activity, the substrate final concentrations were: 5 μM / 12.5 μM / 25 μM / 50 μM / 125 μM / 250 μM / 500 μM. The final concentrations of tamoxifen were: 0 μM / 6.25 μM / 12.5 μM / 25 μM / 50 μM / 100 μM. After incubation at 37 °C for 30 min, 600 ul acetonitrile was added into naloxone incubation reaction system to terminate the reaction by precipitating the protein. The HPLC determined the corresponding metabolites.

To determine whether tamoxifen could inhibit the activity of UGT2B7 in a time-dependent inhibition manner, tamoxifen with a final concentration (0 μM / 6.25 μM / 12.5 μM / 25 μM / 50 μM / 100 μM) was pre-incubated with HLMs (0.1 mg/ml) system including 50 mM Tris-HCl buffer (pH 7.4), 3 mM UDPGA and 25 μg/ml alamethicin for 0 / 15 / 30 / 45 / 60 min at 37 °C. After incubation, 1 μL naloxone (250 μM), 10 mM MgCl2, and 6 mM D-Saccharic acid 1,4-lactone monohydrate were added to the incubation tube. The next experimental steps were as described above.

### Quantification of estrogens using a liquid chromatography-tandem mass spectrometry method

The abundance of estrogens across serum and multiple tissue compartments were assayed as previously described [17]. Estrogens were extracted in dichloromethane at room temperature for 10 min, and estrogen species were analyzed by the LC-MS/MS analysis. The dichloromethane extract was evaporated by Speed-Vac (Thermo Savant, Waltham, MA), and the residue was derivatized using 0.1 M Na_2_CO_3_/NaHCO_3_ buffer and 1 g/L dansyl chloride solution. The chromatographic system consisted of a Shimadzu (California, USA) HPLC system performed on AB Sciex 5500 (Framingham, MA, USA) equipped with an electrospray ionization source (ESI). The chromatographic separation was carried out on a ZOEBAX Eclipse Plus C18 column (2.1 × 50 mm, 1.8 μm) (Agilent Technologies) and protected by a Security Guard (ZOEBAX Eclipse Plus C18, 2.1 × 5 mm, 1.8 μm). The mobile phase (delivered at 0.3 ml/min) consisted of (A) 0.1% formic acid in water and (B) 0.1% formic acid in acetonitrile. Owing to their chemical similarities, d5-E2 is used as the internal standard for the measured estrogens. The MS analysis was performed in positive mode, and the accurate analysis was performed in multiple reaction monitoring (MRM). The lower limit of detection of estrogens was 5pM. Standard curve and linear range of estrogens are listed in Table S2.

### Molecular docking

The crystal structure of the UGT2B7 (PDB ID: 2O6L) and tamoxifen (PDB ID: 6OHU) was obtained from the Protein Data Bank (PDB). Molecular docking was performed between UGT2B7 and tamoxifen to study the binding modes and reveal the most essential residues involved in binding interactions in Sybyl-X2.1 software. The maximum quantity of conformations and the maximum quantity of rotatable bonds were set to the default values: 20 and 100. Additionally, the default optimization of molecules before and after the docking was activated. Other parameters were kept as default values.

### Statistical analysis

Statistical analyses were performed using SPSS Statistics 16.0 software, and all experimental data were expressed as means ± SEM. Differences among multiple groups were tested using one-way analysis of variance followed by Dunnett’s posthoc comparisons. The Student’s t-test tested differences between two groups. Differences were considered significant if *p* < 0.05. Multivariate analysis (Orthogonal Projections to Latent Structures Discriminant Analysis, OPLS-DA) was performed using SIMCA 14.0 software.

## Results

### Estrogen homeostasis profile was disturbed by tamoxifen in experimental rats

**Fig. 2:**
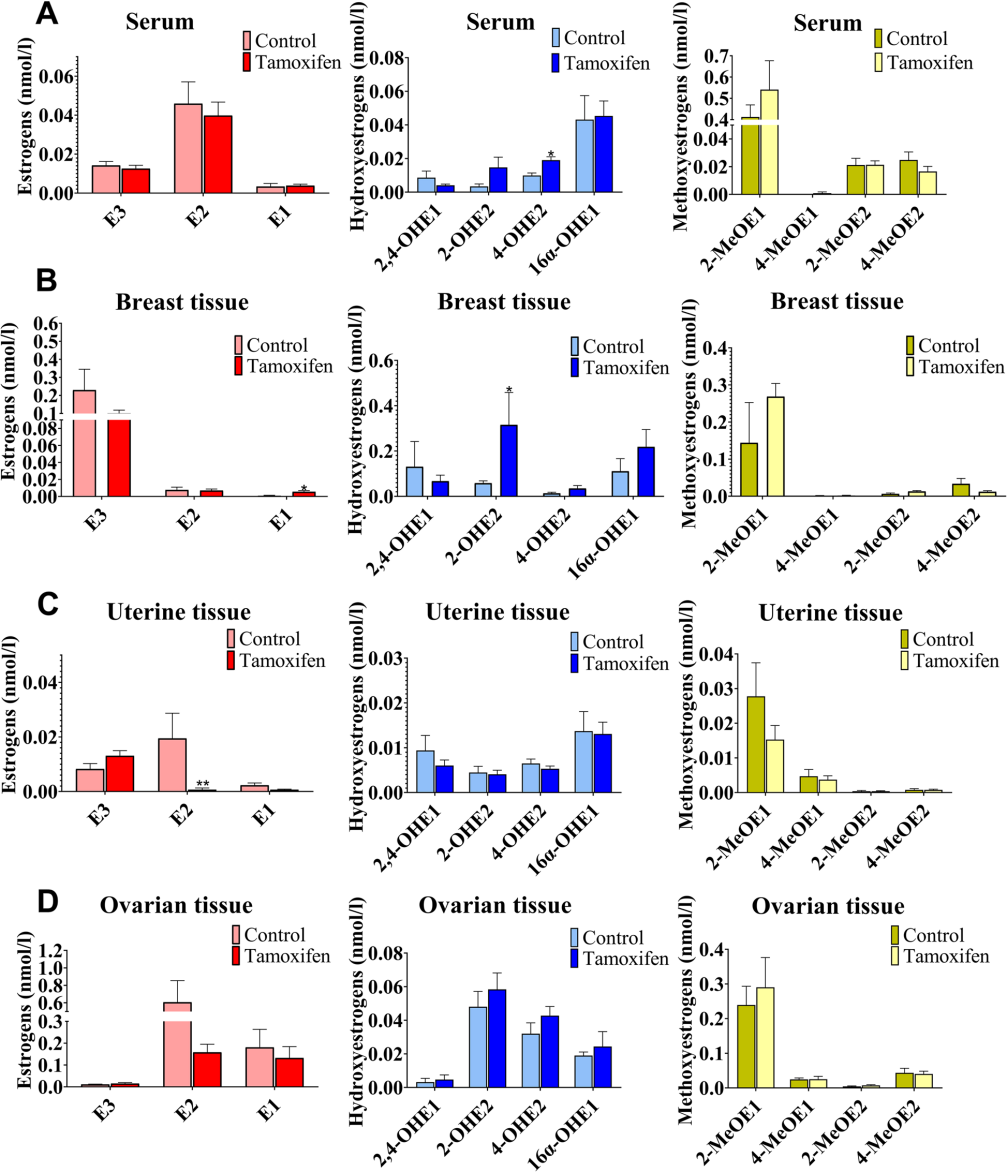
Imbalance of estrogen metabolism in animal models. **A** The effect of tamoxifen on the concentration of estrogen in rat serum. **B** The effect of tamoxifen on the concentration of estrogen in rat breast tissue. **C** The effect of tamoxifen on the concentration of estrogen in rat uterine tissue. **D** The effect of tamoxifen on the concentration of estrogen in rat ovarian tissue. Results are shown as means ± SEM of 7 to 10 rats. *p < 0.05, **p < 0.01 vs control group

Using LC/MS-MS analysis, the concentrations of E1, E2, E3, and 9 estrogen metabolites were measured in rat serum. As shown in Fig. 2A, compared with the control group, the levels of estrogens, especially for 4-OHE2, showed a significantly increasing trend in rat serum tissues of tamoxifen group. Furthermore, the levels of estrogens were examined in breast tissues. As can be observed in Fig. 2B, the levels of estrogens, especially for 2-OHE2 in breast tissues, significantly increased. The estrogens levels in the other necessary estrogen physiological targets, including uterine and ovarian tissue were tested (Fig. 2C, D). Furthermore, we observed that only E2 changed in the uterine tissue, and there was no significant change in the homeostasis of estrogen in the ovarian tissue, indicating that there were tissue differences in the effect of tamoxifen on estrogen homeostasis.

Multivariate analysis (Orthogonal Projections to Latent Structures-Discriminant Analysis, OPLS-DA) was invoked as an unsupervised statistical method to identify potential estrogen homeostatic changes for tamoxifen administration. As shown in Fig. S1, an obvious separation trend can be observed between the control and tamoxifen groups in serum and breast tissues, indicating there was a considerable metabolite difference between the two groups. According to VIP > 1, the metabolites that contributed to the distinction between the two groups were initially screened, and these metabolites were analyzed by t-test (Fig. S1 and Table S3). These results showed that the homeostasis of estrogen homeostasis was affected by tamoxifen at the overall level, mainly by increasing the accumulation of hydroxylated estrogens (2-OHE2, 4-OHE2) in the body, especially in serum and breast tissues, and eventually resulted in serious side effects. Taken together, these results supported the notion that the disturbance of estrogen homeostasis is closely linked to increased risks of side effects of tamoxifen.

### The disorder of estrogen homeostasis was induced by tamoxifen acting on UGT2B

Because the homeostasis of estrogen was closely related to the expression and function of its synthesis and metabolic enzymes, we further studied the effect of tamoxifen on the expression and function of enzymes.

**Fig. 3:**
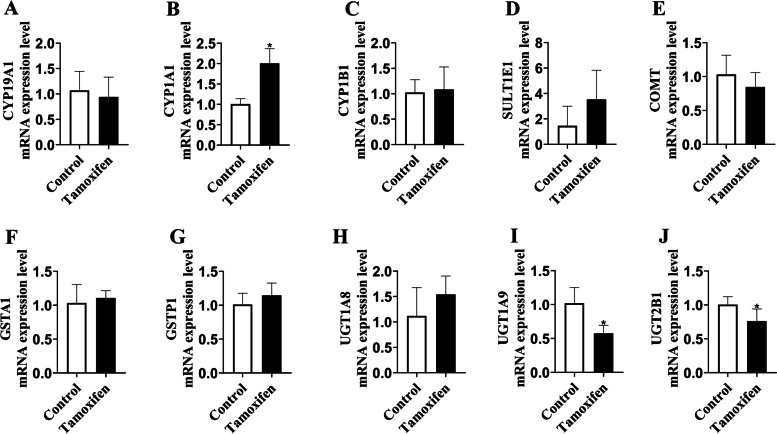
Molecular mechanism of estrogen homeostasis disorder. **A-J** The mRNA expression of CYP19A1, CYP1A1, CYP1B1, SULT1E1, COMT, GSTA1, GSTP1, UGT1A8, UGT1A9, UGT2B1 was detected in liver tissue of 6 rats. Results are shown as means ± SEM of 6 rats. *p < 0.05, **p < 0.01 vs control group

**Fig. 4:**
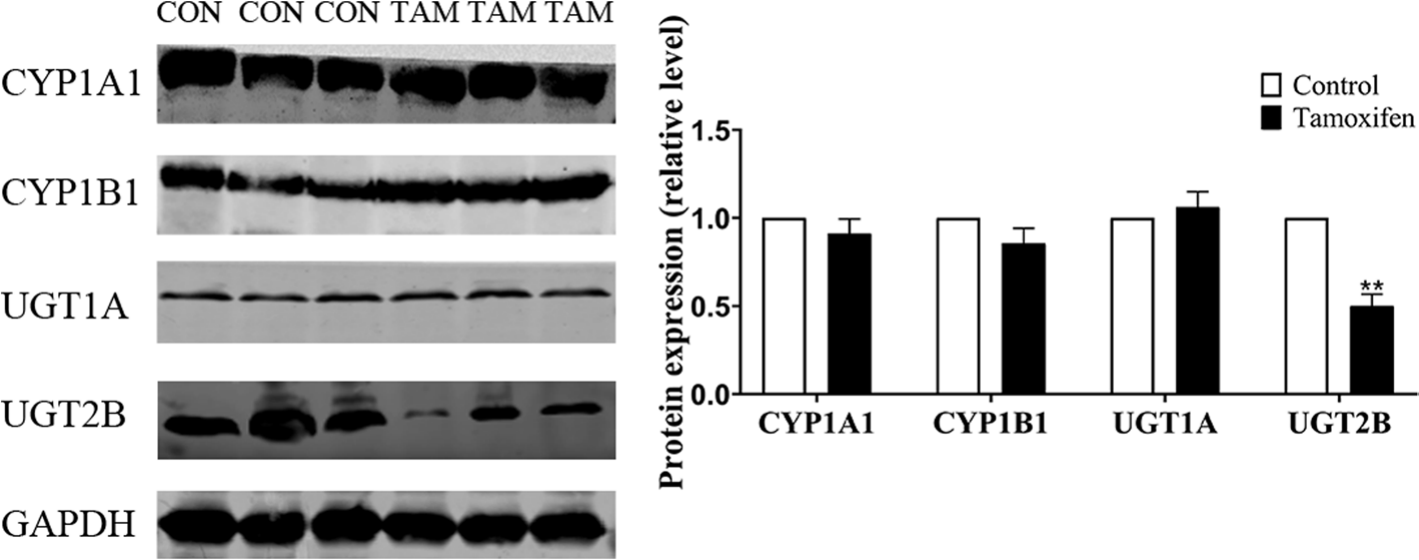
Molecular mechanism of estrogen homeostasis disorder. The protein expression of CYP1A1, CYP1B1, UGT1A and UGT2B was detected in liver tissue of 6 rats. Results are shown as means ± SEM of 6 rats. *p < 0.05, **p < 0.01 vs control group

As shown in Fig. 3 and Table S4, compared with the control group, the expression of CYP1A1 mRNA was significantly increased, and the mRNA expression of UGT1A9 and UGT2B1 (homology with human UGT2B7) was reduced significantly in rat liver tissues of tamoxifen group. Furthermore, using western blotting analysis for further verification, we found that compared with the control group, the expression of CYP1A1, CYP1B1 and UGT1A protein was no significant change, but the expression of UGT2B protein was significantly decreased in rat liver tissue of tamoxifen group (Fig. 4 and Fig. S2). In summary, UGT2B may play an important role in estrogen disorders induced by tamoxifen.

**Fig. 5:**
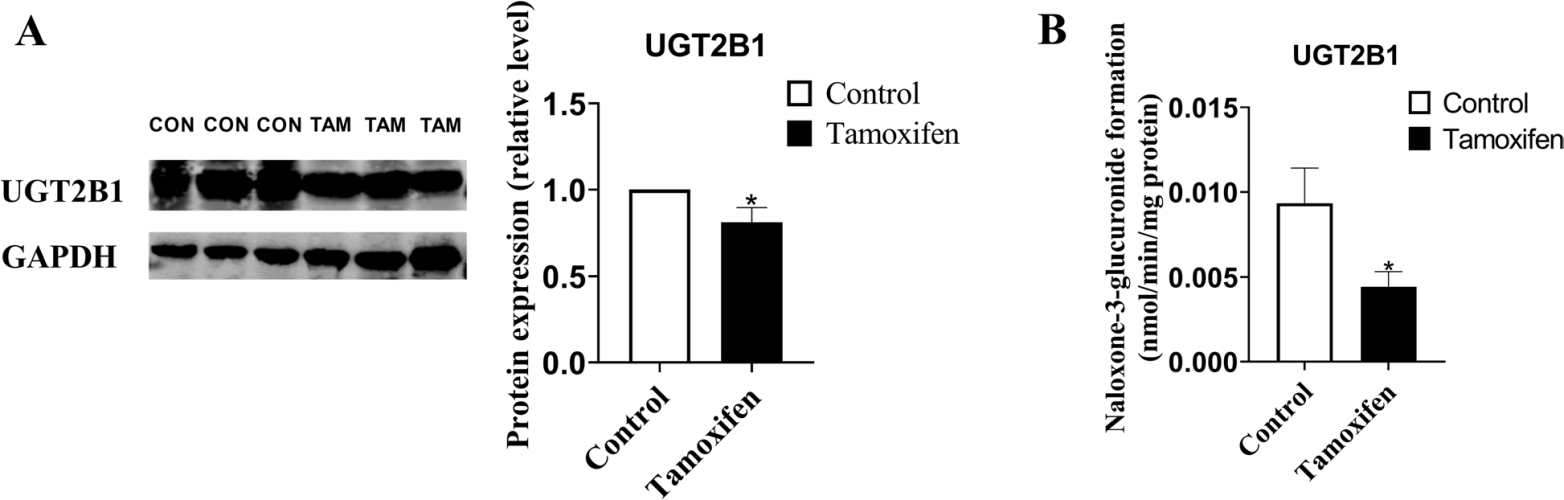
Effect of TAM on the protein expression and activity of UGT2B1 metabolizing enzymes in rat liver tissue. **A** The protein expression of UGT2B1 was detected in liver tissue of 6 rats. **B** Activity of UGT2B1 metabolizing enzymes in rat liver tissue. Results are shown as means ± SEM of 3 rats. *p < 0.05, **p < 0.01 vs control group

Since there was no commercially available rat UGT2B1 antibody, we used UGT2B7 antibody to detect the protein expression of rat UGT2B1 (homology with human UGT2B7). Then, we further examined the protein level of UGT2B1 and found that the expression of UGT2B1 protein was decreased in rat liver tissue of tamoxifen group (Fig. 5A and Fig. S3). In order to explore the effect of tamoxifen on the activities of UGT2B in rats, we extracted S9 from liver tissue of experimental rats and then determined the activities of UGT2B. Naloxone was selected as the specific substrate of UGT2B metabolic enzyme. Our results showed that compared with the control group, naloxone-glucuronide production rate significantly reduced in S9 from rat liver tissue of tamoxifen group, which indicated that the activities of UGT2B enzymes in rats administered with tamoxifen for a period of time were significantly reduced (Fig. 5B). Together, these results suggested that UGT2B expression and activity are primarily decreased under the condition of tamoxifen use, therefore compromising metabolic elimination of estrogens.

### The activity of UGT2B7 was inhibited by tamoxifen in a concentration-dependent manner

**Fig. 6:**
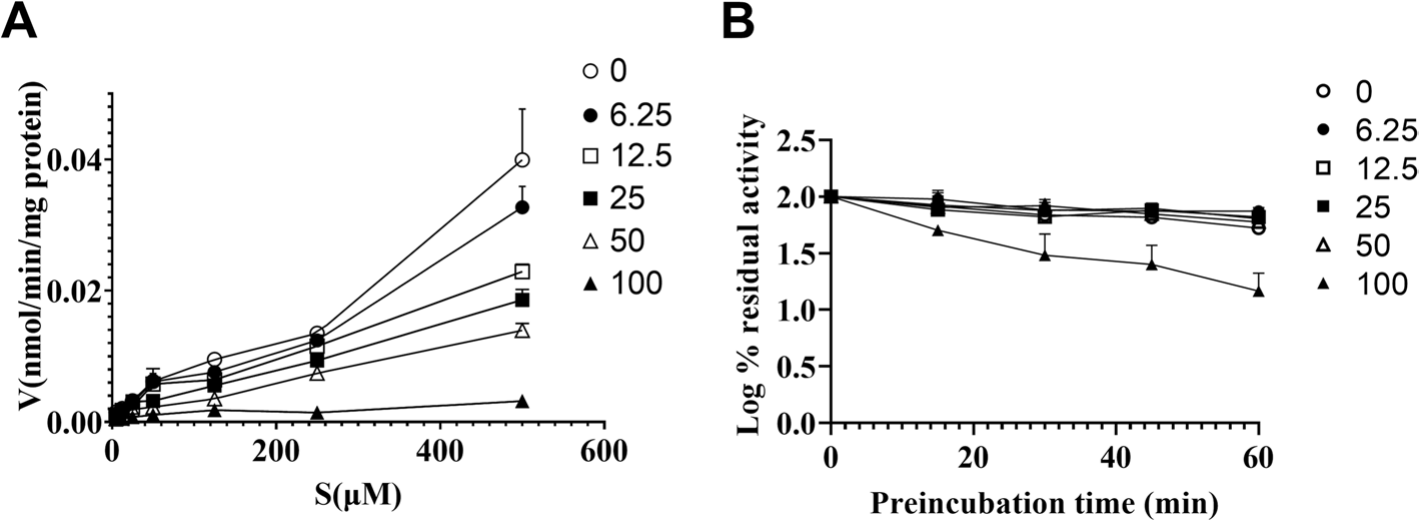
The inhibition of UGT2B7 activity by tamoxifen in HLMs. **A** Concentration-dependent inhibition of UGT2B7 activity by tamoxifen in HLMs. **B** Time-dependent inhibition of UGT2B7 activity by tamoxifen in HLMs. Tamoxifen concentration: ○, 0 μM; ●, 6.25 μM; □, 12.5 μM; ■, 25 μM; △, 50 μM; ▲, 100 μM. Results are shown as means ± SEM

In order to assess how the activity of UGT2B7 was inhibited by tamoxifen, different concentrations of tamoxifen (0 μM / 6.25 μM / 12.5 μM / 25 μM / 50 μM / 100 μM) were incubated with naloxone (5 μM / 12.5 μM / 25 μM / 50 μM / 125 μM / 250 μM / 500 μM) under incubation conditions in HLMs. Of particular interest, as the concentration of tamoxifen increased from 0 μM to 100 μM, the enzymatic reaction rate was significantly reduced, indicating that tamoxifen’s presence inhibited the activity of UGT2B7. In short, tamoxifen is a concentration-dependent inhibitor with a value, K_i_, and IC50 of 37.7 μM and 20.1 μM (Table S5 and Fig. 6A).

Furthermore, in order to investigate whether tamoxifen can inhibit the activity of UGT2B7 in a time-dependent manner, tamoxifen (0 μM / 6.25 μM / 12.5 μM / 25 μM / 50 μM / 100 μM) was incubated under incubation conditions in HLMs for different time (0 min, 15 min, 30 min, 45 min, and 60 min). With the increase of tamoxifen pre-incubation time, the remaining enzyme activity were not decreased significantly (Fig. 6B). When tamoxifen was pre-incubated in HLMs, it could not inhibit the activity of UGT2B7 enzyme and thereby inhibited the metabolism of naloxone, which showed that the UGT2B7 activity was not inhibited by tamoxifen in a time-dependent manner.

### The expression of ligand-activated transcription factors was downregulated by tamoxifen

**Fig. 7:**
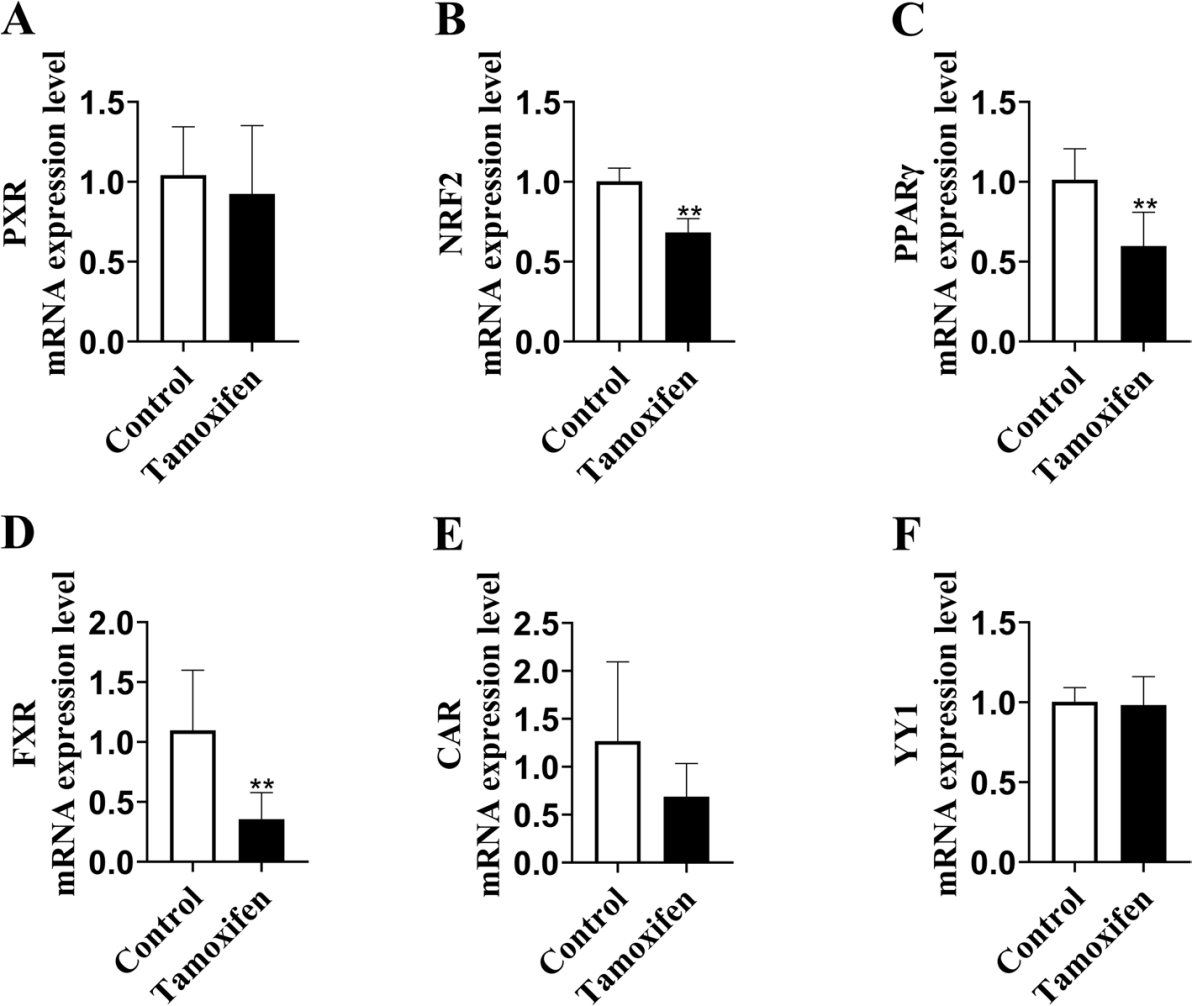
The mRNA expression levels of ligand-activated transcription factors. **A-F** The mRNA expression of PXR, NRF2, PPARγ, FXR, CAR and YY1 was detected in liver tissue of 6 rats. Results are shown as means ± SEM of 6 rats. *p < 0.05, **p < 0.01 vs control group

Emerging evidence has demonstrated that YY1 and ligand-activated transcription factors, such as pregnane X receptor (PXR), NF-E2-related factor 2 (NRF2), peroxisome proliferator-activated receptorγ (PPARγ), Farnesoid X receptor (FXR) and constitutive androstane receptor (CAR) can regulate the transcription of UGT. Therefore, we further extended our research to evaluate the expression levels of these transcription factors in the conditions of tamoxifen use. In accordance with that observed for UGTs, mRNA levels of three transcription factors were significantly decreased in the liver tissue of rats administered with tamoxifen (Fig. 7), suggesting that the imbalance of ligand-activated transcription factors may be an important reason for the decrease of UGT2B expression and activity, which led to the disorder of estrogen homeostasis.

### UGT2B7 docking of tamoxifen

**Fig. 8:**
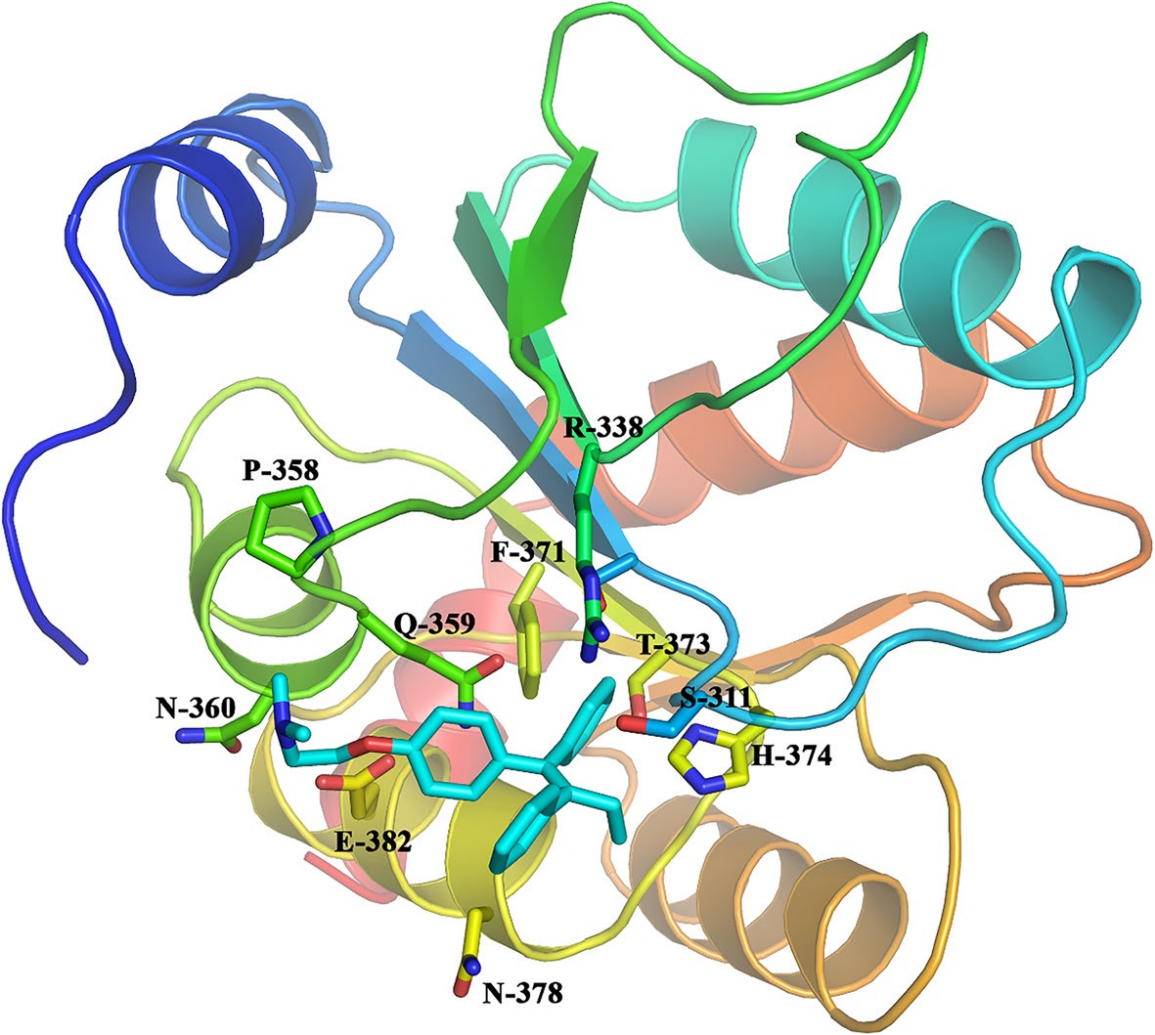
Docking model of tamoxifen and UGT2B7. The structure of tamoxifen is shown as a cyan stick model, the secondary structure of the protein is presented as cartoon drawing and the key amino acid residues of the active site are shown and labeled in a stick model

Molecular docking of tamoxifen as a representative inhibitor to UGT2B7 was performed using the crystal structure of UGT2B7 to study the molecular interaction between UGT2B7 and tamoxifen. As depicted in Fig. 8, tamoxifen could stably bind to the UDPGA binding site of UGT2B7 and interact with multiple amino acid residues, such as hydrophobic interactions with Ser311, Pro358, Gln359, Asn360, Phe371, Thr373, His374, Asn378, and so on. In addition, tamoxifen also had a strong π-π stacking interaction with Phe371 and His374. In conclusion, the hydrophobic and π-π stacking interaction dominated the inhibitory effect of tamoxifen on UGT2B7.

## Discussion

Tamoxifen usually causes a series of side effects, such as hot flashes, endometrial hyperplasia, or even endometrial cancer [24–26] and venous thromboembolic disease [27]. Other side effects of tamoxifen include night sweats, gynecological symptoms (vaginal dryness, vaginal discharge), depression, forgetfulness, sleep changes, weight gain, and decreased sexual function [28–32]. The clinical use of tamoxifen is troublesome due to these side effects. The disorder of estrogen homeostasis in patients may be an essential reason for these side effects [33]. The regulation of estrogen homeostasis depends on the dynamic balance of the production and elimination of estrogen and its metabolites. Metabolizing enzymes in the body is an important means to regulate the dynamic balance of estrogen [34].

According to reports, serum estrogen and tamoxifen levels were positively correlated [35]. During the period of time in postmenopausal women taking tamoxifen, tamoxifen effectively saturated the ER [36]. This may indicate that the importance of estrogen levels may be limited during tamoxifen treatment. However, long-term exposure to tamoxifen had been shown to cause adaptive hypersensitivity to estrogen in breast tumors [37]. We measured the estrogen in the serum and breast tissues of rats that had been taking tamoxifen for one month and found that the content of estrogen, especially hydroxylated estrogens (2-OHE2, 4-OHE2), increased significantly (Fig. 2A, B). These results may indicate that the use of tamoxifen causes a significant increase in estrogen levels in rats. Simultaneously, we observed little difference in estrogen levels in rat ovarian and uterine tissues (Fig. 2C, D). The results showed that this might be due to tissue differences in the effect of tamoxifen on estrogen in vivo. Tamoxifen induced an increase in the concentration of estrogen and disturbed estrogen metabolism to yield excessive catechol estrogen-3,4-quinones, which could increase formation of depurinating estrogen-DNA adducts and the risk of breast cancer. Therefore, it may be important to monitor changes in estrogen levels during long-term tamoxifen treatment [14].

Next, we want to elucidate the underlying mechanism of tamoxifen inducing the disturbance of estrogen homeostasis. CYP450s are the most critical phase I metabolic enzyme that mediates the drugs’ metabolism and other heterologous organisms. The oxidative metabolites of estrogen, especially catechol estrogens, mediated by CYP1A1 and CYP1B1, are then oxidized by a single electron to form reactive semiquinone intermediates, which can directly covalently bind to DNA and eventually lead to DNA damage [38]. Therefore, we detected the mRNA levels of CYP1A1, CYP1B1, and CYP19A1 in rat liver tissues and found that only the mRNA level of CYP1A1 was significantly increased, which may be a factor leading to estrogen disorders (Fig. 3A-C). This is in consistent with previous studies which reported that tamoxifen could upregulate CYP1A1 expression in MCF7 breast cancer cells in an ER-independent manner [39]. However, in our subsequent detection of CYP1A1 protein level, we found no significant change, indicating that CYP1A1 was not a risk factor for tamoxifen to cause estrogen disorders. We discussed the inconsistent trend of RT-PCR and WB results based on this phenomenon, which can be explained by a spatio-temporal interval between the time and site at which transcription and translation of eukaryotic gene expression occur. Simultaneously, the protein level of CYP1B1 was also no significant change, which made us more reason to exclude the participation of CYP1B1 in estrogen homeostasis (Fig. 4). This was inconsistent with the research results of Williams-Brown et al., which may be due to the fact that they used endometrial cancer cells in their research and the administration of estrogen before tamoxifen [40].

Then UGT has attracted our attention. UGT is a phase II drug-metabolizing enzyme located on the endoplasmic reticulum, which increases the water solubility of the substrate and facilitates excretion [41, 42]. UGT-mediated glucuronidation is considered to be a classic detoxification pathway of the body. As with the discussion of the impact of CYP, the mRNA levels of UGT1A8, UGT1A9 and UGT2B1 were detected in rat liver tissues, and the mRNA levels of UGT1A9 and UGT2B1 were significantly reduced (Fig. 3H-J). We speculated that UGT1A9 and UGT2B1 might be significant risk factors for tamoxifen to cause estrogen disorders. In order to verify this conjecture, we continued to detect the protein levels of UGT1A, UGT2B, and UGT2B1 and found that the protein levels of UGT2B and UGT2B1 had a significant decreasing trend (Figs. 4, 5). This indicated that the inhibition of UGT2B may affect the elimination pathway of estrogen, leading to slow elimination of estrogen and the abnormal accumulation of estrogen. Lu et al. reported that UGT2B protein and gene expression levels in most tumor tissues were significantly lower than those in neighboring normal tissues [43]. Numerous clinical trials also proved that the patients carrying mutations UGT2B genotypes might be the best candidate to respond well to tamoxifen treatment to induce effective plasma active tamoxifen metabolite levels [44].

In addition, the quantitative analysis of the enzyme activities of UGT2B showed that the enzyme activities of UGT2B were significantly decreased in rats. In order to determine the main factors affecting the metabolic activity of UGTs, Lu et al. studied the relationship among the metabolic activity, protein, and gene expression levels of each UGT isoform [43]. Compared with other correlations, the metabolic activity ratio of UGT2B was closely related to the reduction ratio of enzyme protein. This close correlation indicated that the reduction of protein expression level might be a prerequisite and plausible mechanism to reduce UGT2B metabolic activity (Fig. 5B). Conversely, the significant impairment of UGT2B function in tissues meant that their translation activity was down-regulated, which may be a susceptible factor for the occurrence and progression of the disease [45]. Therefore, combined with the decrease of mRNA expression and protein level of UGT2B, it can be concluded that the inhibition of UGT2B may be the critical factor of abnormal accumulation of estrogen, which may lead to the occurrence of various adverse reactions and even the progression of breast cancer during the treatment of tamoxifen.

Our data suggested a critical metabolic role of UGT2B in the homeostasis of estrogen metabolism, but the reasons for the decrease in its specific activity were unknow. To verify the regulatory relationship between tamoxifen and UGT2B7, we explored whether there was concentration-dependent inhibition and time-dependent inhibition between tamoxifen and UGT2B7, and found that there was concentration-dependent inhibition of UGT2B7 activity by tamoxifen (Fig. 6), which may be directly responsible for the decrease in UGT2B7 activity. However, it had been shown that all human UGT2B and UGT1A enzymes interacted in different combinations to alter their enzymatic properties. UGT2B7 protein interacted with UGT1A1, UGT1A4, UGT1A6 and UGT1A9 proteins. This interaction affected their enzymatic activities and led to changes in the kinetic parameters of glucuronidation [46]. .Therefore, we need further in vivo systemic studies to determine the interaction of tamoxifen and UGT2B7 in humans [47]. Tamoxifen is metabolized through oxidative reactions catalyzed by a series of cytochrome P450 to its more active derivatives 4-hydroxy-tamoxifen and 4-hydroxy-N-desmethyltamoxifen (endoxifen). Previous studies have identified that endoxifen is as important as, or more important than, 4-hydroxy-tamoxifen to the antiestrogenic action of tamoxifen [48]. The N-desmethyltamoxifen is formed by an oxidative demethylation reaction catalyzed by CYP3A4/5. N-desmethyltamoxifen is further metabolized in the reaction catalyzed by CYP2D6 to form endoxifen. Tamoxifen and its primary metabolites undergo extensive oxidation, mainly through CYP3A and CYP2D6, to metabolites with a series of pharmacological effects. The variable activity of these P450s, caused by genetic polymorphisms and drug interactions, may change the balance of tamoxifen effects in vivo [49]. A large number of studies showed that various tamoxifen metabolites may have different intensity of effect on the induction and inhibition of enzymes. It has been reported that 4-hydroxy-tamoxifen induced CYP3A4, and endoxifen inhibited hSULT2A1, CYP2C8 and CYP2C9 [20, 50]. It can be seen that tamoxifen metabolites may play an important role in regulating the function of metabolic enzymes. Regrettably, our data did not indicate which specific tamoxifen metabolite inhibited the activity of UGT2B. Therefore, in our future study, we will also examine the effects of various tamoxifen metabolites on UGT2B inhibition and correlate specific metabolites with the expression of drug metabolizing enzymes in vivo.

UGTs have been extensively studied for their role in drug and xenobiotic metabolism. Beyond the detoxification of foreign compounds, UGTs also play essential roles in the metabolism of endogenous molecules. This is particularly true for UGT2B, which is involved in the metabolism of estrogen. It has been reported that UGT2B could glucuronidate estrogens and catechol estrogens more efficiently than any other human UGT isoform so as to efficiently bind and metabolize estrogen [51]. Any alteration in estrogen homeostasis is susceptible to participate in the development of pathological situations. In this context, the appropriate regulation of the UGT2B gene is required to avoid the expression from occurring in the wrong place at the wrong moment. The tight control that a series of ligand-activated transcription factors exerts on the activity of UGT genes allows an optimized level of expression in a time-, tissue-, and cell-specific manner [52]. Here, we detected the mRNA levels of ligand-activated transcription factors in liver, and our data showed that three transcription factors were significantly decreased in the liver tissue of rats administered with tamoxifen (Fig. 7). In addition, it has been shown that factors controlling UGT gene expression in the liver include liver-rich transcription factors, several members of the nuclear receptor family, aryl hydrocarbon receptors, and transcription factors involved in the stress response [53].To further explore the binding modes between UGT2B7 and tamoxifen, we performed a molecular docking study to elucidate mechanism through which UGT2B7 expression and activity were inhibited by tamoxifen. Our results showed that the hydrophobic and π-π stacking interaction dominated the inhibitory effect of tamoxifen on UGT2B7 (Fig. 8). This is the first study indicating a direct link to the role of tamoxifen in UGT2B7 expression to the best of our knowledge. This may be a new research target for tamoxifen in pharmacokinetic studies.

## Conclusions

In summary, the homeostasis of estrogen was significantly regulated by tamoxifen, especially the abnormal accumulation of hydroxylated estrogen in the body, which may be closely related to the adverse reactions of tamoxifen. The function and activity of UGT2B was inhibited through hydrophobic and π-π stacking interactions, leading to abnormal regulation of UGT2B and disturbance of estrogen homeostasis, thereby causing adverse effects of tamoxifen. These findings provide a new experimental basis for the rational use of tamoxifen that could facilitate further investigation of its use in the clinical setting.

## Supplementary Information

**Additional file 1.**

**Additional file 2.**

## Data Availability

The data that support the findings of this study are available from the corresponding author upon reasonable request.

## Abbreviations

E1: Estrone
E2: Estradiol
E3: Estriol
2-OHE1: 2-hydroxyestrone
2-OHE2: 2-hydroxyestradiol
2-MeOE1: 2-methoxyestrone
2-MeOE2: 2-methoxyestradiol
4-OHE1: 4-hydroxyestrone
4-OHE2: 4-hydroxyestradiol
4-MeOE1: 4-methoxyestrone
4-MeOE2: 4-methoxyestradiol
16α-OHE1: 16α-hydroxyestrone
UDPGA: UDP-glucuronic acid
CYP: Cytochrome P450
UGT: UDP-Glucuronosyltransferase
PXR: Pregnane X receptor
NRF2: NF-E2-related factor 2
PPARγ: Peroxisome proliferator-activated receptorγ
FXR: Farnesoid X receptor
CAR: Constitutive androstane receptor
