# Supplementary material for: The inhibition of tamoxifen on UGT2B gene expression and enzyme activity in rat liver contribute to the estrogen homeostasis dysregulation
Source: BMC Pharmacol Toxicol. 2022 May 31;23:33. doi: 10.1186/s40360-022-00574-6 (PMC9158366; doi:10.1186/s40360-022-00574-6)
Supplement: Supplementary file 1 — Additional file 1. [file 40360_2022_574_MOESM1_ESM.docx]

**Supplemental Information**

**The Inhibition of Tamoxifen on UGT2B Gene Expression and Enzyme Activity in Rat Liver Contribute to the Estrogen Homeostasis Dysregulation**

Zhixiang Hao^2#^, Jiahao Xu^1#^, Han Zhao^1^, Wei Zhou^1^, Zhao Liu^2^, Shiqing He^2^, Xiaoxing Yin^1^, Bei Zhang^3^, Zhongjian Wang^1^, Xueyan Zhou^1^*

1. Jiangsu Key Laboratory of New Drug Research and Clinical Pharmacy, College of Pharmacy, Xuzhou Medical University, Xuzhou, China.

2. Department of Thyroid and Breast Surgery, the Affiliated Hospital of Xuzhou Medical University, Xuzhou, China.

3. Department of Obstetrics and Gynecology, Xuzhou Central Hospital, Xuzhou Clinical School of Xuzhou Medical University, Xuzhou, China.

^#^ These authors contributed equally to this work.

***Corresponding Author:**

**Prof. Xueyan Zhou**, Jiangsu Key Laboratory of New Drug Research and Clinical Pharmacy, College of Pharmacy, Xuzhou Medical University, 209 Tongshan Road, Xuzhou 221004, China. **E-mail:** zxy851107@xzhmu.edu.cn

**
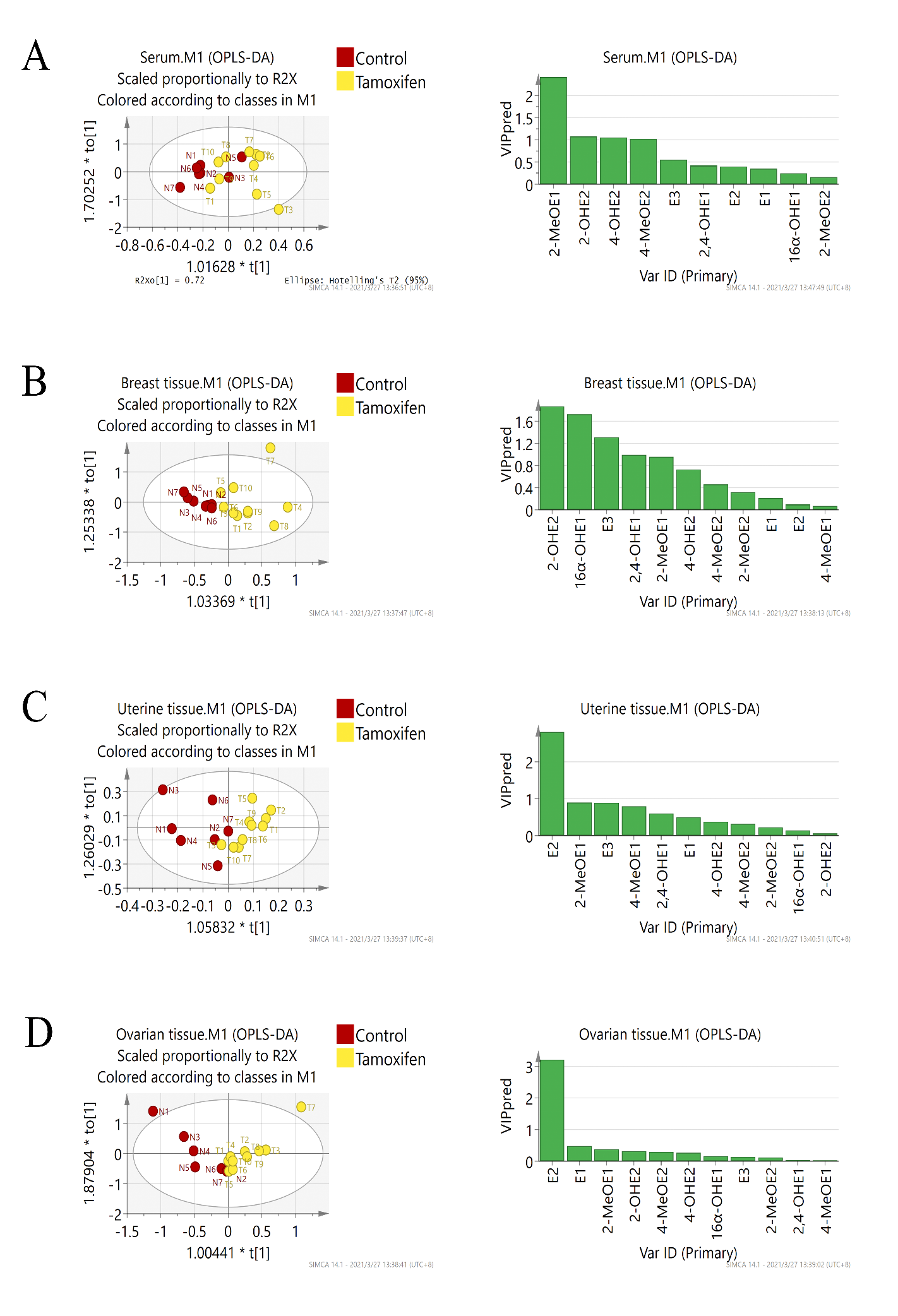
Supplemental Figures:**

**Fig. S1 Imbalance of estrogen metabolism in animal models.** (A) Orthogonal Projections to Latent Structures-Discriminant Analysis (OPLS-DA) score plots and VIP value of estrogen-active substances in serum samples. (B) Orthogonal Projections to Latent Structures-Discriminant Analysis (OPLS-DA) score plots and VIP value of estrogen-active substances in breast tissue samples. (C) Orthogonal Projections to Latent Structures-Discriminant Analysis (OPLS-DA) score plots and VIP value of estrogen-active substances in uterine tissue samples. (D) Orthogonal Projections to Latent Structures-Discriminant Analysis (OPLS-DA) score plots and VIP value of estrogen-active substances in ovarian tissue samples. Results are shown as means ± SEM of 7 to 10 rats. *, p < 0.05, **, p < 0.01 vs control group.


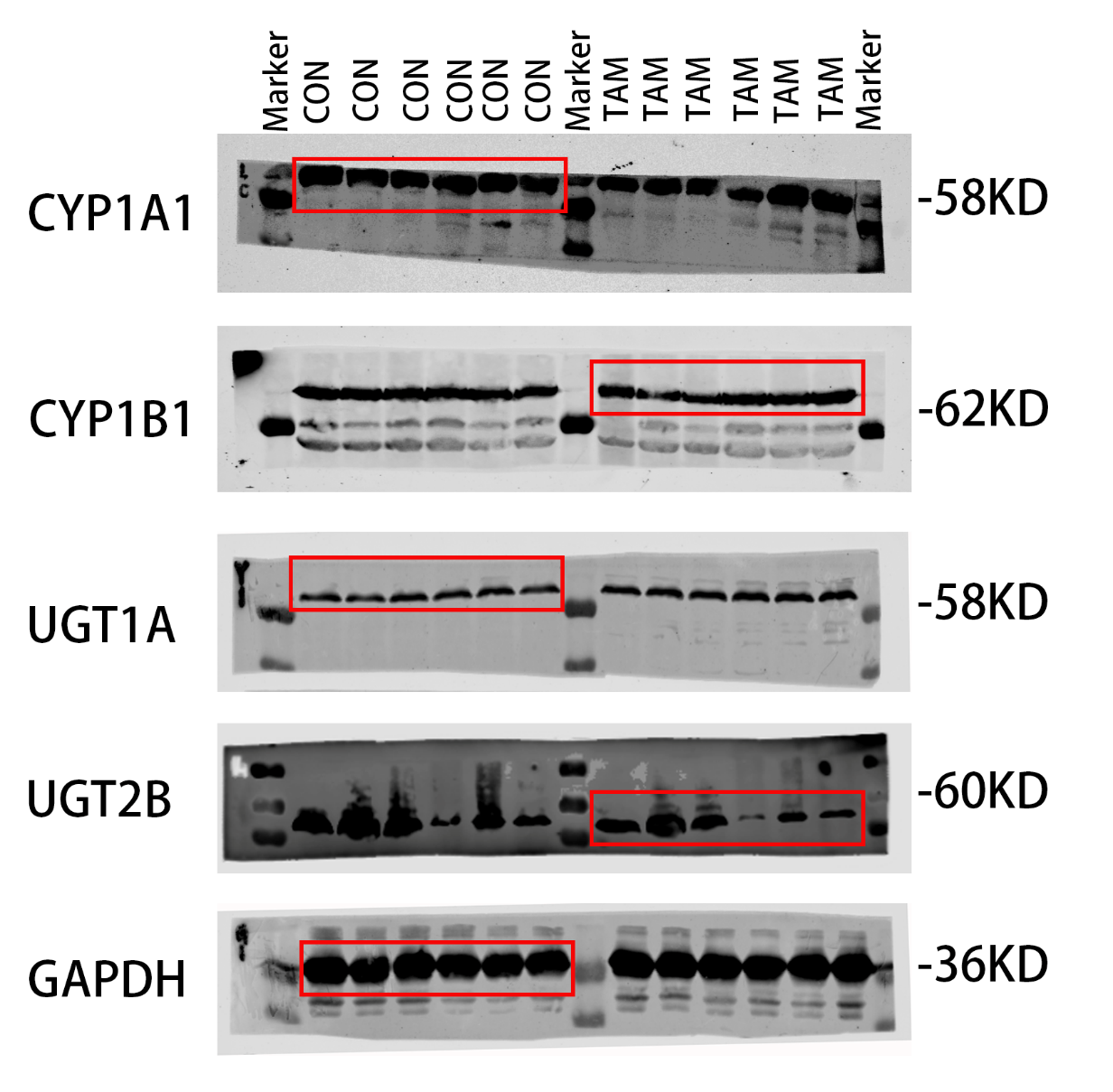


**Fig. S2 Molecular mechanism of estrogen homeostasis disorder.** The protein expression of CYP1A1, CYP1B1, UGT1A and UGT2B was detected in liver tissue of 6 rats.


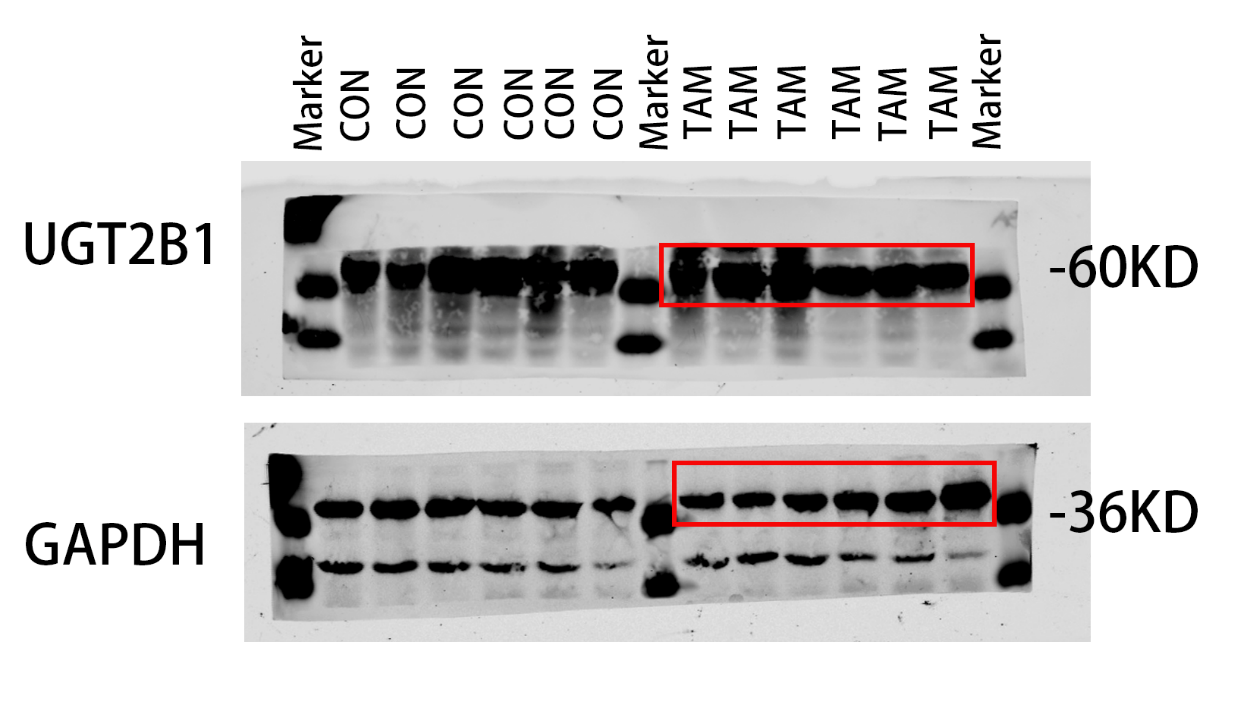


**Fig. S3 Effect of TAM on the protein expression in rat liver tissue.** The protein expression of UGT2B1 was detected in liver tissue of 6 rats.
