# Supplementary material for: The inhibition of tamoxifen on UGT2B gene expression and enzyme activity in rat liver contribute to the estrogen homeostasis dysregulation
Source: BMC Pharmacol Toxicol. 2022 May 31;23:33. doi: 10.1186/s40360-022-00574-6 (PMC9158366; doi:10.1186/s40360-022-00574-6)
Supplement: Supplementary file 2 — Additional file 2. [file 40360_2022_574_MOESM2_ESM.docx]

**Supplemental Information**

**The Inhibition of Tamoxifen on UGT2B Gene Expression and Enzyme Activity in Rat Liver Contribute to the Estrogen Homeostasis Dysregulation**

Zhixiang Hao^2#^, Jiahao Xu^1#^, Han Zhao^1^, Wei Zhou^1^, Zhao Liu^2^, Shiqing He^2^, Xiaoxing Yin^1^, Bei Zhang^3^, Zhongjian Wang^1^, Xueyan Zhou^1^*

1. Jiangsu Key Laboratory of New Drug Research and Clinical Pharmacy, College of Pharmacy, Xuzhou Medical University, Xuzhou, China.

2. Department of Thyroid and Breast Surgery, the Affiliated Hospital of Xuzhou Medical University, Xuzhou, China.

3. Department of Obstetrics and Gynecology, Xuzhou Central Hospital, Xuzhou Clinical School of Xuzhou Medical University, Xuzhou, China.

^#^ These authors contributed equally to this work.

***Corresponding Author:**

**Prof. Xueyan Zhou**, Jiangsu Key Laboratory of New Drug Research and Clinical Pharmacy, College of Pharmacy, Xuzhou Medical University, 209 Tongshan Road, Xuzhou 221004, China. **E-mail:** zxy851107@xzhmu.edu.cn

**Supplemental Table:**

**Table S1. Primer sequences for qRT-PCR (rat)**

| Genes | Sequence |
| --- | --- |
| CYP1A1 | ACCAGGCGAGAAGGTGGATATGAC TGGAGATGCTGAGGACCAGAAGAC |
| CYP1B1 | GAGAGTTGGTGGCAGTGTTGGTG CTCGGCATCGTCGTGGTTGTAC |
| CYP19A1 | AGCCTGCGGTATCAGCCTGTC  AGCCTGTGCATTCTTCCGATGTTC |
| SULT1E1 | GCCGAAATGCCAAAGATGTCGTC  CCAGGAACCATACGGAACTTGCC |
| COMT | CTTGACCACTGGAAAGACCGCTAC  TCTCACATACGCCAGGAAGTCAGG |
| GSTA1 | GTGCAGACCAGAGCCATTCTCAAC  TCATCCAGATCCGCCACTCCTTC |
| GSTP1 | AGCTGGAAGGAGGAGGTGGTTAC  AGCGACCCAGGTGCCTCAAG |
| UGT1A8 | TCTGGACCTGGCTGTGTTCTGG GAAGACCACCGTCAACACGATGG |
| UGT1A9 | AGGCTGTCCATCCTCCCTTC CATGCCCTCTCTGGAGGAGT |
| UGT2B1 | AAAAGTGCTAGTGTGGCCCAC GCCGAAGATACAAGAACCGTGA |
| NRF2 | CATTTGTAGATGACCATGAGTCGC  TCCTGCCAAACTTGCTCCAT |
| PXR | CAAGGGCGTCATCAACTTCG  GGAAGCCGCCATTAGGGTC |
| PPARγ | GCATCAGGCTTCCACTAT  CTTCAATCGGATGGTTCTTC |
| FXR | CGTCCTATTATTCCAACCT  ATTCGCCTGAGTTCATAG |
| CAR | CATATCTCACTCAACACTACG  GACCGCATCTTCCATCTT |
| β-actin | CCCATCTATGAGGGTTACGC  TTTAATGTCACGCACGATTTC |

**Table S2. Standard curve and linear range of estrogens**

| Components | Calibration equation | R2 | Linear range (pM) |
| --- | --- | --- | --- |
| E1 | y=0.187x+0.00118 | 0.9952 | 5-5000 |
| E2 | y=0.73x+0.00638 | 0.9941 | 5-5000 |
| E3 | y=0.182x+0.000681 | 0.9952 | 5-5000 |
| 2-MeOE1 | y=0.2x+0.00184 | 0.9948 | 5-5000 |
| 4-MeOE1 | y=0.134x+0.00228 | 0.9935 | 5-5000 |
| 2-MeOE2 | y=0.189x-0.00056 | 0.9914 | 5-5000 |
| 4-MeOE2 | y=0.432x+0.00582 | 0.9940 | 5-5000 |
| 2-OHE2 | y=0.00962x+9.347e-005 | 0.9925 | 5-5000 |
| 4-OHE2 | y=0.0174x+0.000127 | 0.9947 | 5-5000 |
| 16α-OHE1 | y=0.134x+0.00353 | 0.9933 | 5-5000 |
| 2/4-OHE1 | y=0.0307x+0.000293 | 0.9934 | 5-5000 |

**Table S3. Statistical analysis results of quantitative data of estrogen active substances in rat serum, breast, uterus, and ovarian tissue samples**

|  | OPLS-DA(VIP > 1) | *T* test（*P <* 0.05 or *P <* 0.01 or *P <* 0.001） |
| --- | --- | --- |
| Serum | 2-MeOE1、4-MeOE2、2-OHE2、4-OHE2 | 4-OHE2 |
| Breast tissue | 2-OHE2、16α-OHE1、E3 | E1、2-OHE2 |
| Uterine tissue | E2 | E2 |
| Ovarian tissue | E2 | No difference |

**Table S4. Influence of TAM on mRNA levels (rat).** The mRNA expression of CYP1A1, CYP1B1, CYP19A1, SULT1E1, COMT, GSTA1, GSTP1, UGT1A1, UGT1A9, UGT2B1 was detected in breast tissue of 6 rats. Results are shown as means ± SEM of 6 rats. **p* < 0.05, ***p* < 0.01 vs Control group.

| Metabolism Phases | Gene | Control group | | Tamoxifen group | |
| --- | --- | --- | --- | --- | --- |
|  |  | Mean | SD | Mean | SD |
| Phase I  mono-oxygenases | CYP1A1 | 26.6 | 0.2 | 25.6^*^ | 0.1 |
|  | CYP1B1 | 30.9 | 0.6 | 30.7 | 0.7 |
|  | CYP19A1 | 30.7 | 0.6 | 30.7 | 0.4 |
| Phase II transferases | SULT1E1 | 32.0 | 1.3 | 30.4 | 0.9 |
|  | COMT | 15.7 | 0.4 | 15.9 | 0.2 |
|  | GSTA1 | 15.8 | 0.3 | 15.6 | 0.3 |
|  | GSTP1 | 22.8 | 0.2 | 22.5 | 0.3 |
|  | UGT1A8 | 17.3 | 0.4 | 16.5 | 0.3 |
|  | UGT1A9 | 24.9 | 0.2 | 25.7^*^ | 0.2 |
|  | UGT2B1 | 18.2 | 0.2 | 18.5^*^ | 0.2 |

**Table S5. Kinetic parameters for naloxone metabolism by Human liver microsomes.**

| Parameters | Value |
| --- | --- |
| *Ki* (μM) | 37.7 |
| IC50 (μM) | 20.1 |
